# Imaging the Limbic System in Parkinson’s Disease—A Review of Limbic Pathology and Clinical Symptoms

**DOI:** 10.3390/brainsci12091248

**Published:** 2022-09-15

**Authors:** Magdalena Banwinkler, Hendrik Theis, Stéphane Prange, Thilo van Eimeren

**Affiliations:** 1 Multimodal Neuroimaging Group, Department of Nuclear Medicine, Faculty of Medicine and University Hospital Cologne, University of Cologne, 50937 Cologne, Germany; 2 Department of Neurology, Faculty of Medicine and University Hospital Cologne, University of Cologne, 50937 Cologne, Germany; 3 Institut des Sciences Cognitives Marc Jeannerod, CNRS, UMR 5229, Université de Lyon, 69675 Bron, France

**Keywords:** PET, SPECT, MRI, ventral striatum, amygdala, hypothalamus, cingulate, hippocampus, impulse control disorders, depression

## Abstract

The limbic system describes a complex of brain structures central for memory, learning, as well as goal directed and emotional behavior. In addition to pathological studies, recent findings using in vivo structural and functional imaging of the brain pinpoint the vulnerability of limbic structures to neurodegeneration in Parkinson’s disease (PD) throughout the disease course. Accordingly, dysfunction of the limbic system is critically related to the symptom complex which characterizes PD, including neuropsychiatric, vegetative, and motor symptoms, and their heterogeneity in patients with PD. The aim of this systematic review was to put the spotlight on neuroimaging of the limbic system in PD and to give an overview of the most important structures affected by the disease, their function, disease related alterations, and corresponding clinical manifestations. PubMed was searched in order to identify the most recent studies that investigate the limbic system in PD with the help of neuroimaging methods. First, PD related neuropathological changes and corresponding clinical symptoms of each limbic system region are reviewed, and, finally, a network integration of the limbic system within the complex of PD pathology is discussed.

## 1. Introduction

Parkinson’s disease (PD) is a frequent and multisystem neurodegenerative disease, affecting the human central, peripheral, and enteric nervous system. Neuropathology is characterized by intraneuronal inclusions containing misfolded α-synuclein aggregates, called Lewy bodies. The pathophysiology is primarily characterized by a dopamine deficiency due to a progressive loss of dopaminergic neurons innervating the basal ganglia. This pathophysiology is associated with the cardinal motor manifestations and some of the cognitive dysfunctions observed in PD [1,2,3].

Due to the prominent motor manifestations in PD, for a long time, the main research focus has primarily been set on the pathophysiology of the motor system. Consequently, in the past several decades, there has been remarkable progress in the understanding of the functional organization of the motor system and most notably how pathological changes in the basal ganglia result in motor abnormalities [4]. However, conceptualizing PD as just a disorder of the nigrostriatal dopaminergic system is reductionistic, and it is now well established that the pathophysiology of PD is far more widespread and complex [5]. Histological examination has revealed that damage to the nigrostriatal pathway is accompanied by extensive extranigral pathology. Besides dopaminergic neurons in the motor system, nerve cells of the limbic system have shown to be vulnerable to destruction. In fact, the limbic system and its connections are subject to major pathological changes in the course of the disease [6,7,8]. For instance, the amygdala, a core limbic structure, harbors dense Lewy pathology in PD patients, which starts as early as Braak stage 3, i.e., around the same time than the substantia nigra [9,10]. Since the limbic system is integral for emotions, learning, and memory, there has been increasing recognition that changes in this system substantially contribute to the symptom complex which characterizes PD, ranging from neuropsychiatric to vegetative and cardinal motor dysfunction.

### 1.1. What Is the Limbic System?

The limbic system describes a complex of brain structures central for memory, goal-directed, affective, and emotional behavior. The concept of the limbic system has been highly influential in the field of neuroscience and can look back on a long history. Over time, the entity to what the term “limbic system” refers to underwent substantial changes, but the concept persists to the present day [11]. The term “limbic” stems from the Latin word “limbus” which means “border” and was first used in 1664 by the physician Thomas Willis to denote the curved cortical border around the brainstem [12]. When Broca spoke of the “Great Limbic Lobe”, he thought of it as a primarily olfactory structure [13], and it was not until the mid-20th century that Papez and McLean associated the limbic system with emotional functioning in humans [14].

Despite many efforts, to this date, consensus regarding the structures that form the limbic system is still lacking. Most commonly, regions of the limbic system encompass the cingulate gyrus, hippocampus, parahippocampal gyrus, amygdala, mammilary bodies, hypothalamus, as well as the nucleus accumbens [15,16], with dense intrinsic connections [17]. Historically, the ventral striatum was not always seen as an integral part of the limbic system, despite early recognition of the strong reciprocal connections of the ventral striatum and all major limbic structures. However, the ventral striatum, which encompasses the nucleus accumbens, is a central hub for the connection between the motor and limbic system [18]. Therefore, and due to its importance in PD, we decided to include the ventral striatum in the present review and emphasize its role within the limbic system and in neuropsychiatric manifestations related to PD.

Regardless of the absence of a clear definition, the limbic system constitutes a functional concept, and is characterized by dense afferent projections from brainstem and forebrain nuclei, which contributes to behavioral modulation. From this functional standpoint, the limbic system is seen as essential for the regulation of emotional behavior. In fact, the limbic system is a center which links stimuli with social, emotional, or motivational relevance to a set of behavioral outputs; thus, by linking the internal and external world, it controls appropriate behavioral responses [19]. A previous published theory stated that there might not be one limbic system, but rather separate, independent circuits for emotions and memory centered either on the amygdala or the hippocampus [11].

### 1.2. Imaging Changes

With the progression of PD, limbic structures become increasingly affected by pathology, causing injury to the nerve cells, eventually resulting in cell death [6,20]. Neuroimaging provides insight into these biological changes, which can be due to the underlying neuropathological mechanisms as well as compensatory responses to the disease. To gain a better understanding of the PD related changes of the limbic system, as well as the relationship between changes and clinical presentation, in vivo structural and functional neuroimaging is key, including single photon emission computed tomography (SPECT), positron emission tomography (PET), and novel magnetic resonance imaging (MRI) techniques [21], which, remarkably, were not systematically reviewed to the best of our knowledge.

### 1.3. Aim of Review

Comprehensive reviews which focus on the pathophysiology of motor impairments in PD exist [4,22], but, to our knowledge, there is currently no review which provides an overview of limbic neuropathological changes and their related clinical manifestations. For this reason, this systematic review puts the spotlight on the limbic system in PD and gives an overview of the most important structures affected by the disease, their function, disease-related alterations, as well as corresponding clinical symptoms. In this scope, we provide an overview of neuroimaging changes (i.e., MRI, PET and SPECT) and symptoms related to limbic alterations in PD. Furthermore, we develop the network integration of the limbic system within the complex of PD pathology and highlight the role of the ventral striatum. In particular, we seize the suggestion of Rolls [11] concerning two separate limbic systems dedicated to emotion or memory. We transfer these ideas to the cognitive decline in PD, especially PD dementia, and the typical neuropsychiatric symptom complex such as depression, apathy and impulse control disorders (ICD) and how they interact with the motor disturbance in PD.

## 2. Methods

### 2.1. Literature Search and Selection Strategy

A systematic literature search was performed in PubMed. Based on the Medical Subject Headings (MeSH) terms provided by the National Library of Medicine and basic literature on the limbic system, we selected the most important regions of the limbic system. These regions included the amygdala, hippocampus, hypothalamus, cingulate gyrus, substantia innominata and septal nuclei, fornix and mammillary bodies, habenula and pineal body, and ventral striatum.

For each limbic region of interest (ROI), we conducted an independent literature search by using the following search query: (Parkinson’s disease OR Parkinson) AND (LIMBIC_ROI) AND ((imaging) OR (MRI) OR (PET) OR (SPECT) OR (fMRI) OR (functional MRI)). The searches were limited to human studies only and because we wanted to focus on the most recent literature, studies published between 2018 and 2022 were included. Due to the methodological challenges related to the resolution limits of SPECT, PET and MRI for small, millimetric structures, neuroimaging studies are scarce for the fornix and mammillary bodies as well as the habenula and pineal body [23]. As a consequence, the publication time period was extended, and all available publications were considered for these ROIs. Eligibility for inclusion was determined by predefined criteria.

#### 2.1.1. Inclusion Criteria

A study was included if it met the following criteria:

- English language;
- Parkinson’s disease;
- Use of neuroimaging methods (i.e., PET, MRI, SPECT);
- Human study.

#### 2.1.2. Exclusion Criteria

A study was excluded if it met the following criteria:

- The limbic ROI was not explicitly examined in the study;
- Review paper or case report;
- Not accessible and not freely available.

### 2.2. Limbic Parkinson’s Disease Wordcloud

Additionally, we generated a word cloud representing the 200 most discriminant words in the abstracts with ‘Parkinson’s disease’ in the title and containing the word ‘limbic’. Specifically, we extracted all nouns and adjectives in 508 abstracts containing ‘limbic’ and in a random sample of 2500 abstracts (out of 61,565) not containing ‘limbic’ for articles entitled ‘Parkinson’s disease’ extracted from PubMed between 1977 and March 2022. Stopwords, proper and verbal nouns, and adverbs were filtered out using the Text Mining package and a custom word list following annotation using udpipe in R. This resulted in two corpuses of 4979 and 16,179 words, respectively, with a total of 32,522 and 141,216 occurrences, respectively. Words occurring in at least five abstracts containing ‘limbic’ were then selected, representing 1052 words with 30,130 and 66,452 occurrences, respectively. We then calculated the Youden’s index (also known as bookmaker informedness) for each word, summarizing the performance for a given word to correctly classify an abstract into a given corpus. By analogy, with a diagnostic test, a value of 1 indicates a perfect test, meaning that there are no wrongly classified abstracts using this word, with no false positives or false negatives. Height is proportional to the informedness value.

## 3. Results

**Figure 1:**
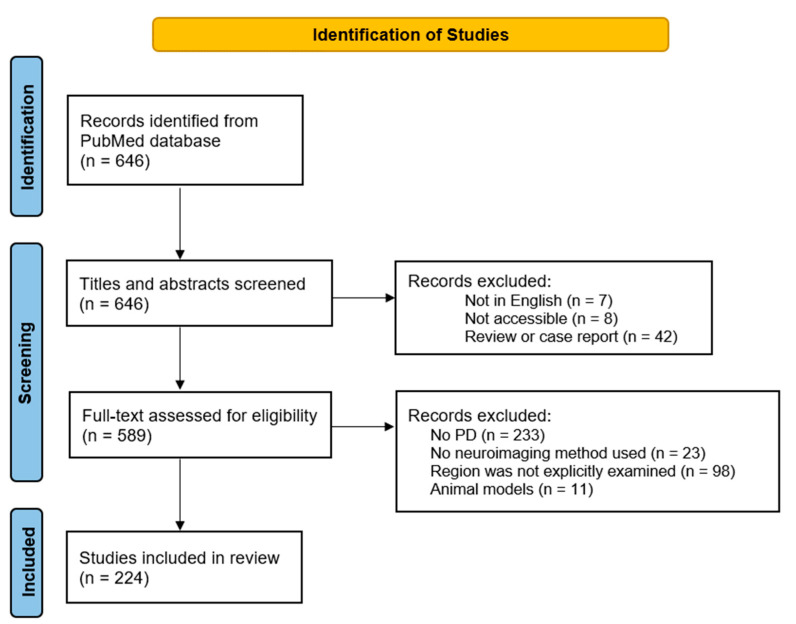
Preferred reporting items for systematic reviews and meta-analyses (PRISMA) flow diagram.

**Figure 2:**
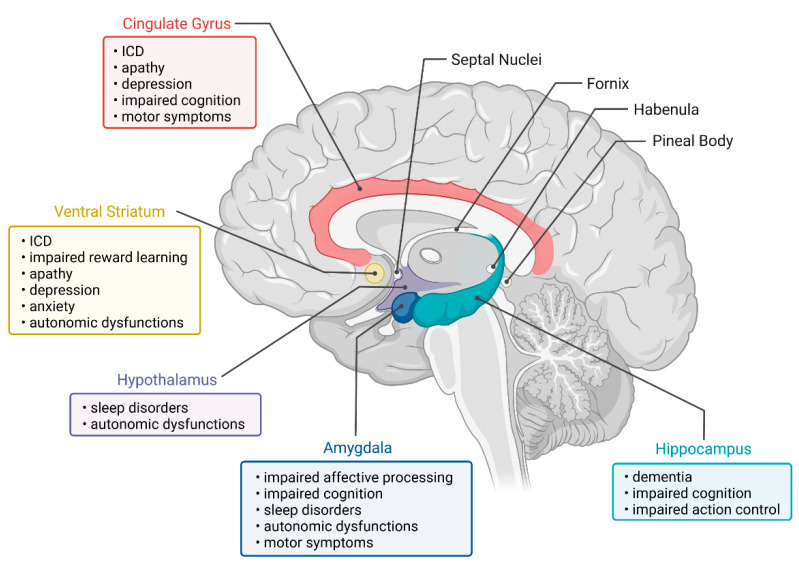
Representation of selected limbic regions affected by PD pathology and their associated clinical symptoms. Created with Biorender.com.

The combined search result of all limbic regions can be seen in Figure 1. Our electronic database search generated a total of 646 studies, of which 224 were identified as relevant and were included in the review. We extracted the following information for each study: key components of general study information (title, author, year, and journal), and study characteristics (main research interest, neuroimaging method, sample size, and outcome). This information is summarized in Supplementary Table S1. In this paper, we first review each limbic region by providing an overview of its anatomy, function, and pathology, and by discussing the most recent evidence for structural and functional imaging and the relationship with PD symptoms. An overview of selected limbic structures and their corresponding PD symptoms can be found in Figure 2. Finally, we conclude and integrate this new information into the limbic system model proposed by Rolls [11].

**Figure 3:**
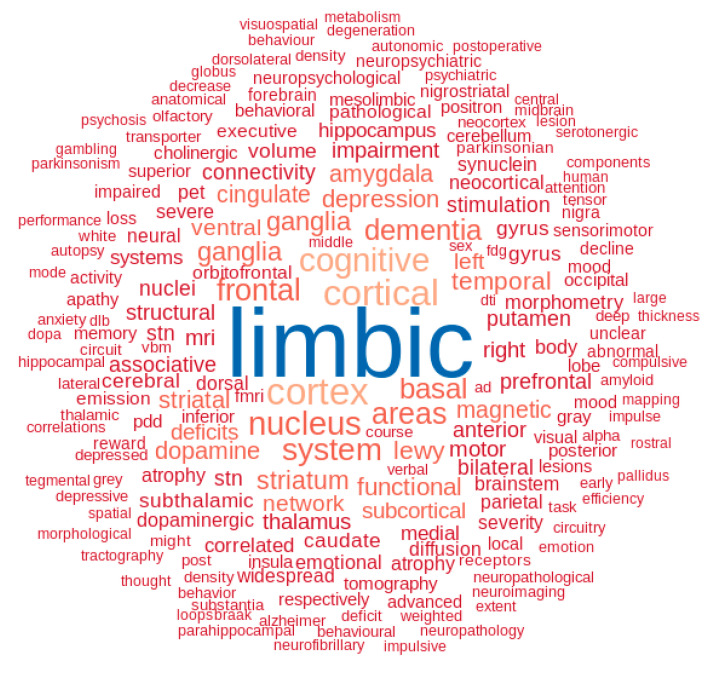
A cloud of the limbic system. The 200 most discriminant words characterizing the abstracts containing the word ‘limbic’ are depicted in the wordcloud. This illustrates the network organization of the limbic system, involving the frontal and cingular cortico-striatal system, its modulation by dopaminergic, serotonergic, noradrenergic and cholinergic projections from brainstem and forebrain nuclei, and highlights the critical role of the limbic system in dementia, depression, apathy and impulse control disorders.

In addition, we performed a data-driven, text mining analysis of all abstracts containing the word ‘limbic’ since 1977 in order to provide an unbiased account of the key components of the limbic system in Parkinson’s disease (Figure 3). This illustrates the network organization of the limbic system, involving the frontal and cingular cortico-striatal system and its modulation by brainstem and forebrain small nuclei and their dopaminergic, serotonergic, noradrenergic and cholinergic projections, subjected to multiple pathological processes. Symptoms and disorders involving the limbic system, including memory and dementia, apathy, depression, impulse and reward-related disorders are highlighted.

## 4. Regions of the Limbic System

### 4.1. Amygdala

#### 4.1.1. Anatomy and Function

The amygdala is an almond shaped structure nestled deep in the medial temporal lobe. It is a highly differentiated region composed of distinct subareas or nuclei; thus, this is sometimes also referred to as the amygdala complex. One widely acknowledged description divides the amygdala into a phylogenetically primitive group of nuclei, which are associated with the olfactory system (central, medial, cortical, and nucleus of the lateral olfactory tract), and a phylogenetically newer group of nuclei (basal and lateral nucleus) [24].

Information about the external environment is transmitted from the sensory thalamus and sensory cortices to the amygdala. In turn, the amygdala has reciprocal connections with the midline and orbital prefrontal cortex, hippocampus, and sensory areas. Unidirectional outputs encompass the striatum, nucleus accumbens, and the bed nucleus of the stria terminalis, which are involved in translating the input signals into behavioral outputs [25,26]. Most notably implicated in emotion and motivation, the amygdala makes essential contributions to the processing of fearful and rewarding environmental stimuli. It is suggested that the amygdala subserves incentive learning, a process by which stimuli are attributed affective significance, and thereby motivates behavioral responses and actions [27].

#### 4.1.2. Pathology

Severe pathological changes can be observed in the amygdala during the progression of PD. Misfolded proteins are detected early in the amygdala and, according to the Braak PD staging scheme, the amygdala is affected in stage 3 [9,28]. However, the amygdala is not uniformly affected by the pathology. Lewy bodies and Lewy neurites exhibit a specific distribution, with some nuclei undergoing prominent changes and others remaining largely uninvolved. Early and strongly affected nuclei are the central and accessory cortical nucleus [7,10].

#### 4.1.3. Neuroimaging Evidence in PD

##### Neuropsychiatric Symptoms

Numerous studies have identified reduced gray matter volume in the amygdala [29,30,31,32]. While there is inconsistency regarding amygdala atrophy especially in early PD, it becomes more pronounced with increasing disease duration and severity [33,34,35,36,37,38]. A great body of evidence has revealed a significant relationship between PD-related changes in the amygdala and affective traits. For example, abnormal activation within the amygdala has been observed during affective processing [39], and fMRI studies have found over-activation of the amygdala to be associated with psychotic symptoms and anxiety [40,41]. A study examining early-stage PD patients found no association between the structural covariance of the amygdala and the severity of anxiety symptoms. Thus, it seems that the amygdala-to-whole-brain structural covariance might not be affected early on [42]. However, anxiety levels of PD patients did positively correlate with the functional connectivity between the amygdala and the superior parietal lobule as well as the *weighted degree* (the sum of functional connectivity strengths in a specific brain area with all other brain areas) of the left amygdala [43]. Additionally, anxiety was also associated with a smaller amygdala volume [44] and with a lower dopaminergic binding in females [45]. Furthermore, abnormal connectivity between the amygdala and hippocampus has been related to depression [46].

Molecular imaging studies using [^11^C]DASB PET report alterations in the serotonergic system in the amygdala starting already in the preclinical stage [47,48] and evidence of [^18^F]FPEB PET and [^18^F]FDG PET studies further reveal significantly upregulated glutamate receptors as well as hypometabolism in the amygdala of PD patients [48,49,50,51]. In addition, PD patients with ICD had reduced D2/3 receptor binding in the ventral striatum and putamen. In this context, PD patients with ICD exhibited a positive correlation between midbrain and amygdala D2/3 binding [52].

##### Cognitive Symptoms

Neuroimaging studies have demonstrated a consistent association between hippocampal volume loss and dementia [53]. However, recent evidence also suggests involvement of the amygdala, whereby PD patients with cognitive impairment display even greater amygdala atrophy compared to patients without cognitive impairment [54]. Furthermore, another study reported hypoactivation of the amygdala in PD patients off medication versus healthy controls when generating a successful response in a choice reaction time task [55].

##### Motor and Other Symptoms

Changes in the amygdala may also contribute to the cardinal motor dysfunction [55]. In this context, a study was able to accurately predict Unified Parkinson’s disease rating scale (UPDRS) III scores by using a sparse set of connectivity features, including the putamen and amygdala [56]. Additionally, amygdala mean diffusivity was positively associated with the UPDRS scores for non-motor symptoms as well as activities of daily living impairment, indicating that amygdala changes may affect movement through the regulation of affective states [57]. Dysfunction of the amygdala has further been demonstrated through affected nodal centrality [58,59] and, in line with the diverse functions of the amygdala, studies also report impaired olfaction, sleep disturbances, and autonomic dysfunction in combination with amygdala alterations [60,61].

Regarding connectivity changes, PD patients have demonstrated enhanced coherence of the white matter tract in the amygdala−accumbens−pallidum pathway which can be interpreted as dysfunctional hyperconnectivity [62] and the amygdala to midbrain functional connectivity was found to be modulated by dopamine agonists [63]. Decreased connectivity with the cerebellum has been noted [64], and the white matter structural connectivity showed greater disruption in males compared to females [65]. Increased amygdala connectivity with the putamen and decreased connectivity with the frontoparietal network has been related to freezing of gait, suggesting an increased striato-limbic load in combination with reduced top-down attentional control [66]. Decreased connectivity with the inferior parietal lobule, lingual gyrus, and fusiform gyrus were linked to the severity of hyposmia and cognitive performance [67]. Furthermore, higher D2-like binding in the amygdala was associated with better stopping control [68]. This superior inhibitory control in subjects with higher D2-like binding may indicate limbic regulation of motor control.

##### Conclusions

In conclusion, PD-related changes in the amygdala are not only linked to alterations in affective processing such as anxiety, but a more extensive symptom complex including cognitive performance, sleep disorders, autonomous symptoms, and cardinal motor dysfunction.

### 4.2. Hippocampus

#### 4.2.1. Anatomy and Function

The hippocampus is located bilaterally within the medial temporal lobe and its shape grossly resembles a seahorse, which inspired its naming [69]. The hippocampal formation comprises four distinct parts: Cornu ammonis (hippocampus proper), dentate gyrus, entorhinal area, and subiculum. Hippocampus proper and the dentate gyrus form together the C-shaped rings and the hippocampus proper is further subdivided into CA1, CA2, CA3, and CA4 [70]. Decades of research on hippocampal function have established its critical role in learning and memory processes and the link between hippocampal damage and amnesic symptoms [71,72]. In this respect, the hippocampus is critically involved in the formation of new declarative memories [73], as well as spatial navigation involving place and grid cells [74,75]. In addition, the hippocampus also excerpts an influence on the hypothalamic-pituitary-adrenocortical activity and emotional behavior with close reciprocal connections with the amygdala [70].

#### 4.2.2. Pathology

Neuropathological studies have played a critical role in uncovering involvement of the hippocampus in the pathophysiology of PD and indicate increasing α-synuclein deposition that is associated with significant neuronal dysfunction [76,77]. According to Braak staging, the hippocampus harbors significant pathology from stage 4 onwards [9,28]. Accumulating evidence suggests hippocampal involvement not only in dementia but also in motor dysfunctions and other neuropsychiatric aspects of PD [78].

#### 4.2.3. Neuroimaging Evidence in PD

##### Cognitive Symptoms

Cognitive dysfunction is one of the most prevalent and debilitating non-motor symptoms of PD and 20 years into the disease, and more than 80% of PD patients will develop dementia [79]. In face of this detrimental symptom, extensive research has addressed pathological changes in the hippocampus. Morphological studies have reported reduced hippocampal volume in PD patients, whereby atrophy has inconsistently been reported in early-PD but becomes more pronounced with increasing disease duration and is strongly linked to cognitive decline including memory, spatial working memory, and language impairments [29,31,36,37,54,60,80,81,82,83,84,85,86,87,88,89,90,91,92,93,94,95,96,97]. The severity of volume loss has also been found to be predictive of conversion to dementia [53,98,99]. Additionally, PD-related alterations in iron content [61,100,101], texture [34], microstructural integrity [102,103,104], arteriolar-cerebral-blood-volume [105], connectivity [106,107,108], but not synaptic density [109] have been reported in combination with reduced cognitive performance. Serotonergic binding in the hippocampus of PD patients was not associated with cognitive performance [110]. In addition, serotonin transporter (SERT) loss extended to the hippocampus in PD patients with the A53T mutation in the SNCA gene (associated with autosomal dominant development of PD), but not in premotor carriers [48].

##### Neuropsychiatric Symptoms

As mentioned earlier, changes in the hippocampus do not only result in cognitive deficits but manifest in other nonmotor symptoms. Hyposmia and sleep disturbance have been associated with hippocampal dysfunction [60,92,111,112,113,114,115] and modulation of the parasympathetic outflow seems to be impaired as well [116]. In addition, decreased left hippocampal volume and altered functional connectivity were reported in depressed individuals [46,117]. Degeneration of the hippocampus was found in combination with psychotic symptoms, which manifest as visual or minor hallucinations, whereby psychosis severity could be predicted from hippocampal volumes [118]. The development of psychotic symptoms was linked to increased signaling in the hippocampus, amygdala, striatum, and the dopaminergic midbrain [41]. Furthermore, the underlying involvement of visual illusions include not only the primary visual cortex and surrounding regions, but also the hippocampus [119]. Finally, dopamine depletion is also linked to attenuated reward signaling in the mesolimbic system, and deficient reward-related processing in the hippocampus has been shown to be partially restorable through the administration of dopaminergic medication [120].

##### Motor and Other Symptoms

Dysfunction of the hippocampus has recently also been linked to movement dysfunctions [57,121,122]. Patients experiencing freezing of gait, demonstrated reduced activation of the hippocampus and decreased connectivity with the cerebellum relative to controls [123,124,125]. Higher behavioral impairment scores were related to increased connectivity of the hippocampus with the right caudate head, which may represent a compensatory mechanism [126]. Overall, hippocampal connectivity seems to be more strongly affected in males than in females [65]. Interestingly, a 6-week ‘exergaming’ intervention (combination of a motivating and visually stimulating computer game with physical exercises) reported a significant volume increase in the left hippocampus, suggesting hippocampal volume changes in PD patients can be induced by non-pharmacological interventions [127]. Furthermore, the role of the mesocorticolimbic dopaminergic system in action control has been highlighted by [^18^F]fallypride PET imaging studies, which demonstrated overall reduced binding in PD, but a significant association between faster response inhibition and greater D2-like binding potential in the hippocampus, thereby advocating limbic regulation of the action-control network [52,68].

Reciprocal regulation of dopamine and glutamate has been noted in the nigrostriatal, mesocortical, and mesolimbic system, circuits which are affected by PD pathology. In this context, a study suggests that glutamate is more than 20% upregulated in several mesocortical regions, including the hippocampus, amygdala, and putamen [50]. Furthermore, a specific spatial covariance pattern of the serotonergic system was reported in PD, which comprises decreased binding in the putamen, caudate, and substantia nigra and preserved binding in the hypothalamus and hippocampus. Expression of this pattern was more strongly in PD compared to healthy controls and significantly correlated with disease duration [47,128].

##### Conclusions

In summary, the hippocampus of PD patients demonstrates substantial volume loss with increasing disease duration, which is strongly linked to cognitive impairment and dementia. However, multiple studies have also highlighted its regulatory role in action control.

### 4.3. Hypothalamus

#### 4.3.1. Anatomy and Function

Situated at the base of the brain with a size of just 4 cm^3^, the hypothalamus constitutes one of the smallest and phylogenetically most conserved parts of the human brain. It is located below the thalamus and above the midbrain. Anteriorly, it is bounded by the optic chiasm, laterally by the optic tracts, and posteriorly by the mammillary bodies. Despite its small size, the anatomy of the hypothalamus is complex, constituting a collection of several distinct nuclei which are commonly organized into the anterior, tuberal and posterior (mammillary) region [129,130,131].

The functional roles played by the hypothalamus are manifold. It regulates vital functions including thirst, hunger, sleep, temperature, mood, circadian and seasonal rhythms, sex drive as well as the production of some of the body’s essential hormones. In the broadest sense, its role is an integrative one. It maintains homeostasis by bringing together sensory and bodily information and accordingly activating endocrine, autonomic, and behavioral responses [130,132].

#### 4.3.2. Pathology

PD related pathological changes in the hypothalamus were noted decades ago by numerous researchers, including Lewy himself [133,134]. These changes include the presence of Lewy bodies, specifically in the tuberomammillary and posterior hypothalamic nuclei, as well as the loss of dopamine. According to the Braak staging scheme, the PD-associated pathology targets hypothalamic nuclei in stage 4, thus in the early symptomatic phase [9,28].

#### 4.3.3. Neuroimaging Evidence in PD

##### Autonomous Symptoms and Sleep Disturbance

Recent neuroimaging evidence complements early findings. A high-resolution MRI study of >360 participants found no significant difference in the hypothalamus volume of PD and non-PD individuals—thereby implying that the histopathologically detected involvement of the hypothalamus in PD is not observable as global hypothalamic atrophy, and further suggesting that the macrostructure of the hypothalamus remains rather stable throughout the disease course [135]. In line with the regulatory role of the hypothalamus in the autonomic nervous system, reduced hypothalamic functional connectivity with the thalamus and striatum was observed in PD patients with a higher burden of autonomic symptoms compared to those with a lower burden [136]. Furthermore, excessive daytime sleepiness, a common autonomic symptom in PD, was associated with increased phosphodiesterase 4 (PDE4) expression in brain regions that are involved in sleep regulation, including the hypothalamus. PDE4, an intracellular enzyme expressed in neurons and glial cells, is inter alia implicated in the modulation of dopaminergic activity [113,137]. Loss of SERT was also noted in the hypothalamus in symptomatic and premotor A53T SNCA carriers [48] and a recent [^11^C]DASB PET study has linked reduced serotonergic function in the hypothalamus to sleep dysfunctions in PD patients [138]. However, lower hypothalamic SERT binding was not observed in early disease stage PD patients [47,128].

##### Conclusions

To conclude, in accordance with the regulatory role of the hypothalamus, PD-related changes in this region mainly manifest as alterations in autonomic functions.

### 4.4. Cingulate Gyrus

#### 4.4.1. Anatomy and Function

Anatomically, this brain region spans the corpus callosum and is therefore called “cingulum”, which is the Latin word for belt. It was described by Broca in 1877 as a part of the so-called “grand lobe limbique” [139]. The cingulate cortex can be divided into four regions which are again divided into subregions: the anterior cingulate (subgenual and pregenual), the midcingulate (anterior and posterior), the posterior cingulate (dorsal and ventral) and the retrosplenial cortex [140,141]. Among others, the anterior cingulate receives input from the orbitofrontal cortex (OFC), the amygdala, the parahippocampal gyrus, and it projects to other parts of cingulate cortex, the medial prefrontal cortex and to the striatum [142]. The anterior cingulate cortex (ACC) is involved in action-outcome learning, based on the integration of a prediction error signal by taking into account whether an event was expected or not [142,143]. In addition, the subgenual cingulate cortex also integrates the emotional component of reward [144]. The posterior cingulate cortex receives input from the temporal lobe and projects to the hippocampus and has been linked to the spatial component of episodic memory [142]. Furthermore, the posterior cingulate cortex (PCC) is a key structure of the so-called default-mode-network, which is active when an individual is at rest. According to Pearson, this region might be responsible for detecting changes of the environment during rest [145].

#### 4.4.2. Pathology

In PD, the cingulate cortex is affected in the Braak stage 5 [28].

#### 4.4.3. Neuroimaging Evidence in PD

##### Neuropsychiatric Symptoms

The cingulate cortex has a central role in ICD in PD, mainly involving connectivity changes of the cingulate cortex to other brain regions. Severity of ICD negatively influenced the connectivity between accumbens and ACC [146], whereas another study showed an increase in reward-related connectivity between these two regions, which was independent of dopaminergic medication [63]. A reduced between-network but increased within-network connectivity of the salience network was found in PD with ICD [147]. Apart from connectivity changes, the severity of ICD correlated positively with the volume of the subgenual ACC [146]. Concerning sex differences, greater atrophy in the cingulate was reported for men than women with PD [65]. In addition to the ACC, the PCC is also involved in ICD [146]. In particular, in hypersexual PD patients under dopamine replacement therapy, excessive wanting of reward lead to heightened blood-oxygen-level-dependent (BOLD) activity in the PCC [148].

Similar to the ventral striatum, the ACC is not only involved in ICD and reward processing but also plays an important role in other neuropsychiatric symptoms. Structural imaging revealed a volume reduction of the ACC [149] as well as reduced white matter integrity in PD patients with depression [149,150,151]. An fMRI study revealed that the anterior cingulate might be a hub region for depression in PD [152]. In this regard, increased connectivity between the ventral tegmental area and the ACC was reported in depressive as compared to non-depressive PD patients [153], in addition to network dysfunction of the PCC in depressive PD patients [154]. Furthermore, apathy was also associated with reduced neuronal activity [155], microstructural alterations [156] and an increase in amyloid depositions in the ACC [157], whereas anxiety was related to thinning of the cingulate cortex [44]. In turn, functional connectivity between the anterior cingulate and the temporo-parietal junction was positively correlated with a higher quality of life in PD [158]. Furthermore, PET studies have reported serotonergic dysfunction in the cingulate of PD patients [159,160,161,162]. Notably, increased serotonergic innervation in the ACC and ventral striatum was recently demonstrated in PD patients who were apathetic at diagnosis and reverted apathy under dopamine replacement therapy, suggesting compensatory plasticity in early PD [163]. To conclude, damage of the ACC during the disease course of PD seems to play an important role for the development of behavioral symptoms.

##### Cognitive Symptoms

Regarding memory and cognition, PD patients with mild cognitive impairment (MCI) had an increased functional connectivity between the posterior cingulate and the thalamus when compared to demented PD patients [164] and fractional anisotropy of PCC bundles correlated positively with cognition [165]. The role of the PCC in cognition and memory was further stressed in functional [108,164,166,167,168] and structural [169] MRI studies.

In addition, lower structural and functional connectivity between the insula and ACC [170,171] and lower functional connectivity between the caudate and ACC was reported in MCI [126]. Reduced connectivity of the ACC to the dorsolateral prefrontal cortex could be ameliorated by an eight-week cognitive training [172]. However, another study found increased connectivity between the insula and middle cingulate in PD with MCI [173]. Cholinergic innervation of the cingulate cortex was associated with cognitive performance in PD [174]. A multimodal study with dopamine transporter (DAT) SPECT and FDG PET demonstrated that a degeneration of the cognitive striatum, as measured by [^123^I]FP-CIT binding ratios, is related to a reduced glucose metabolism in the anterior cingulate [175], which emphasizes the link between PD progression and the development of cognitive deficits.

##### Motor and Other Symptoms

Motor symptoms were linked to imaging changes of the cingulate cortex (e.g., reduced connectivity and metabolism) in particular for hypokinetic symptoms and during motor learning a higher blood flow was reported in the ACC [176]. PD patient with predominant tremor had a higher functional connectivity between the right fronto-insular cortex and the ACC when compared to akinetic-rigid PD [177] and micrographia, a typical early motor symptom, was associated with a reduced glucose metabolism in the middle cingulate gyrus [178]. In PD patients with deep brain stimulation, an impaired drawing ability was related to a reduced perfusion of the cingulate after surgery [179]. Furthermore, a decrease in speech loudness in PD was associated with an increased activation of the anterior cingulate as compared to healthy controls [180].

Regarding sleep disorders, PD patients with rapid eye movement sleep behavior disorder (RBD) had reduced functional connectivity to temporal, frontal, insular and thalamic regions when compared to healthy controls. When compared to PD patients without RBD, PD patients with RBD had a reduced connectivity between posterior cingulate and precuneus [181]. PD patients with pain relieved under deep brain stimulation showed a reduced activity of the anterior cingulate in fMRI as compared to those without relief of pain [182], with a positive correlation between DAT binding in the posterior cingulate and the pain threshold [183]. Furthermore, a meta-analysis of PET studies, which investigated microglia-mediated neuroinflammation via translocator protein levels, found significantly elevated levels in the ACC as well as PCC of PD patients, thus highlighting the disease related dysfunction of the cingulate [184].

##### Conclusions

In sum, the cingulate gyrus is critically involved in neuropsychiatric symptoms related to PD, but also influences motor performance. The ACC seems to be rather associated with behavioral symptoms such as ICD, depression and apathy, in connection with the ventral striatum. Furthermore, altered function of the PCC is more related to cognitive decline, with close connection with the temporal lobe and the hippocampus.

### 4.5. Substantia Innominata and Septal Nuclei

#### 4.5.1. Anatomy and Function

The substantia innominata and septal nuclei (triangular, medial, lateral and dorsal septal nuclei, septofimbrial nucleus, nucleus of diagonal band, nucleus of the anterior commissure) represent telencephalic cortical, predominantly cellular, grey matter regions providing critical cholinergic projections to the amygdala (through the stria terminalis), hippocampus (through the fornix), lateral hypothalamus (through the medial forebrain bundle), habenula (through the stria medullaris thalami) and tegmentum, and receiving major afferents from the amygdala and hippocampus, besides the orbitofrontal, mesiotemporal cortex and insula.

As indicated by its name, anatomical definition of the substantia innominata remains challenging, englobing a collection of cholinergic and non-cholinergic nuclei of the basal forebrain below the anterior commissure within the quadrigone delineated by the anterior perforated substance, and the globus pallidus and ansa lenticularis on each side, in close vicinity to the basal ganglia and amygdaloid complex. Overall, cholinergic neurons are divided into eight groups named Ch1–Ch8, of whom the nucleus basalis of Meynert (nbM, Ch4) gives the chief cholinergic projection to the amygdala and hippocampus, the latter being also innervated by the Ch1 and Ch2 groups. The nbM also project to part of the striatum which receives widespread cholinergic input from the pedunculopontine nucleus (Ch5) and dorsolateral tegmental nuclei (Ch6), also responsible for the cortical cholinergic innervation. As such, the nbM (Ch4), medial septal nucleus (Ch1) and nucleus of the vertical limb of the diagonal band (Ch2) specifically project to the subcortical and cortical limbic system, playing a critical role for attention, memory and arousal.

#### 4.5.2. Pathology

Abundant Lewy bodies are found in the nbM, corresponding to Braak stage 3, with severe accumulation in stage 4 [28], contrasting with often lower density in connected limbic areas [185]. It was postulated that severe extension of Lewy body pathology to the magnocellular nuclei of the basal forebrain (basal nucleus of Meynert, interstitial nucleus of the diagonal band and medial septal nucleus) may represent a prerequisite of neocortical synucleinopathy [28]. Importantly, postmortem studies found consistent cortical cholinergic reduction across PD patients, whereas Ch4 cell loss and hippocampal cholinergic innervation were variable, but strongly depleted in those with PD dementia [76].

#### 4.5.3. Neuroimaging Evidence in PD

##### Neuropsychiatric and Motor Symptoms

Using in vivo MR imaging, no volumetric difference is found for Ch1-2 and Ch4 between patients with PD and healthy controls in early PD [103,186]. However, lower volume is found in more advanced PD [187,188], correlating with cortical thinning in the bilateral posterior cingulate, parietal, and frontal and left insular regions in patients with PD-MCI [189]. This is in line with decreased metabolism in the parietal and occipital cortices [190] and decreased functional connectivity between the nbM and the right superior parietal lobe and postcentral gyrus [191]. In addition, patients with lower substantia innominata volume had decreased connectivity between the caudate and frontal, parietal, temporal, precentral and PCC [192]. Furthermore, decreased myelin content is found in its emerging projections [193] together with lower fractional anisotropy within the frontolateral tracts in PIGD-dominant patients [194]. Altered connectivity between the nbM and parietal and occipital cortex may be implicated in visual hallucinations [195] and grey matter density in the Ch4 group and centromedial amygdala was specifically associated with apathy, but not depression, in PD [196]. In addition, recent studies pinpointed the role of cholinergic forebrain nuclei in gait disorders, with lower nbM volume predicting increased gait variability in patients with early PD [197] and in those undergoing STN-DBS.

##### Cognitive Symptoms

Lower Ch4 density was consistently associated with impaired cognition, including attention and visuospatial dysfunction [198]. Furthermore, free-water using diffusion-weighted imaging is consistently increased in the NbM in those with cognitive impairment at baseline and predicted future cognitive decline together with lower volume [97,199,200,201,202]. However, non-corrected DTI metrics were not related to cognitive impairment in the cholinergic forebrain but in the hippocampus [103]. Lower volume of the nbM also predicted cognitive decline in patients undergoing STN-DBS [203]. Notably, single trajectory DBS targeting both the GPi and nbM did not improve cognition in a recent cross-over trial in 6 PD patients [204], consistent with previous trials in PD [205].

Ch1-2 volume might be greater in PD patients without cognitive impairment compared to those with cognitive impairment and to healthy controls [186]. This may indicate greater resilience, although longitudinal atrophy is observed [200]. Notably, the cholinergic projections of the nBM plays a critical role in cortical activation causing desynchronized EEG pattern in the attentional state. Interestingly, volume of the cholinergic basal forebrain correlated positively with alpha reactivity in PD, whereas it was specifically related to EEG changes in pre-alpha power in people with MCI [206]. Importantly, the role of the bidirectional delta/theta band network between the nbM and inferior and mesial temporal lobe structures including the parahippocampal gyrus was recently highlighted in patients with PD dementia [207].

### 4.6. Fornix and Mammillary Bodies

#### 4.6.1. Anatomy and Function

The fornix represents a small C-shaped projection tract located on each side of the midline, emerging from the flattened fibers of the fimbria, where part of the fibers forms the hippocampal commissure. Thereafter, most of the fibers join, forming a body under the splenium of the corpus callosum, run anteriorly and divide above the interventricular foramen. As such, it connects the hippocampus (subiculum and entorhinal cortex) with the mammillary bodies and anterior thalamic nuclei (postcommissural fibers), and with the septal region (precommissural fibers), through the posterior and anterior columns respectively according to their division around the anterior commissure [208].

In turn, the mammillary bodies send projections in their immediate vicinity to the anterior and dorsal thalamic nuclei (mammillo-thalamic tract, also known as the bundle of Vicq d’Azyr) and to the tegmental nuclei (mammillo-tegmental tract). Importantly, the fornix conveys the cholinergic projections from the septal nuclei to the hippocampus. As such, the fornix and mammillary bodies are part of a hippocampocentric group, critically involved in memory and spatial orientation [208]. Therefore, it is considered as target for DBS in AD. Overall, the fornix is central to the circuit described by Papez and MacLean.

#### 4.6.2. Pathology

Alterations are observed starting in Braak stage 3, although structural alterations of the fornix may be already present in people at risk for PD [209] and be related to peripheral inflammation [210].

#### 4.6.3. Neuroimaging Evidence in PD

Although widespread, white matter alterations were observed in the fornix in patients with MCI [211], advanced stage PD patients with short-term memory impairment [212], and those with excessive daytime sleepiness [213]. Moreover, structural alterations of the fornix using DWI are already found in moderate [214] and early PD [215], as well as in de novo PD patients for whom connectivity of the fornix was associated with hyposmia [216]. In addition, the volume of the fimbria [217] and left hippocampus–amygdala transition area was correlated with visuospatial/executive function in PD patients with MCI [93]. However, decline of the hippocampal formation and fimbria was observed in PD patients progressing to dementia, associated with impairment in the attention and executive domains [99]. This is consistent with altered structural integrity in the fornix and Ch3-Ch4 cholinergic neuronal groups, correlating with impaired Mini-Mental State Examination scores and executive function in early PD. Loss of structural integrity observed in the Ch1-Ch2 groups correlated with the severity of recall memory impairment [104].

In addition, lower fractional anisotropy and higher mean diffusivity were found in the fornix-stria-terminalis in patients with probable RBD [218], possibly restricted to those with concomitant depression [219].

### 4.7. Habenula and Pineal Body

#### 4.7.1. Anatomy and Function

Together with the pineal body, the habenula and habenular commissure forms the posterior division of the diencephalon, named epithalamus. As such, the habenula can be identified as a small triangular area adjacent to the wall of the 3rd ventricle and to the medial surface of the thalamus, extending into the third ventricle with the habenular commissure. The habenula is further divided into a medial and lateral part, which can be visualized using ultra-high field MRI or susceptibility-weighted imaging at 3T [220].

The lateral habenula receives critical limbic afferent projections through the stria medullaris thalami, from the cortex (ACC, anterior insula, dorsal OFC), but also from the hypothalamus, septal nuclei and brainstem monoaminergic nuclei (ventral tegmental area, median raphe and locus coeruleus), and ventral pallidum for its most lateral part [221]. As such, the lateral habenula is deeply integrated within the emotional limbic system, representing a target for DBS for treatment-resistant depression [222]. In turn, the habenula sends efferent projections to the septal nuclei, ventral tegmental area, dorsal raphe nucleus and locus coeruleus, modulating the mesolimbic dopaminergic, serotonergic and noradrenergic circuits. Hence, the habenula represents a major modulator of the reward circuit, critical for the regulation of impulsive behaviors, next to the amygdala.

#### 4.7.2. Pathology

Even though literature on the neuropathology of the epithalamus is sparse, it is assumed that this brain region starts harboring significant PD pathology in Braak stage 4 [9,28].

#### 4.7.3. Neuroimaging Evidence in PD

##### Neuropsychiatric Symptoms

Specifically, dysfunction of the habenula was implicated in PD punding, with lower resting-state functional connectivity between the bilateral habenula and left frontal and precentral cortices [223]. In addition, increased connectivity was observed between the habenula and the thalamus bilaterally, and with the striatum and posterior cingulum in the left hemisphere, in comparison to patients without ICDs. Interestingly, increased connectivity between the amygdala and thalamus and striatum was also observed, highlighting the imbalance of inhibitory control and reward for patients engaging in repetitive behaviors regardless of the lack of reward. Furthermore, patients with punding also had greater severity of apathy and depression relative to healthy controls and patients without ICDs matched for age and disease duration [223]. Interestingly, depressive-like behaviors are observed in Parkinsonian preclinical models related to decreased connectivity between the serotonergic raphe nuclei and the lateral habenula, dentate gyrus of the hippocampus, thalamus and hypothalamus, possibly reversed by dopaminergic treatment [224]. Notably, studies of the habenula remains scarce, which may change with high-resolution imaging using ultra-high field MRI.

The pineal gland is a small neuroendocrine structure derived from the epithalamus and located at the roof of the 3rd ventricle, below the habenular commissure and above the superior colliculus and dorsal to the posterior commissure. Importantly, secretion of small neuropeptides and biogenic amines including melatonin is closely regulated by the anterior hypothalamic nuclei, involving the suprachiasmatic nuclei, considered the central clock within the hypothalamus. Notably, the hypothalamus receives dense projections from the serotonergic midbrain raphe neurons and from the lateral medullary noradrenergic neurons, besides intrinsic dopaminergic neurons. Melatonin is central to the organization of the sleep-wake cycle, and its nychthemeral pattern was found to be dysregulated across neurodegenerative disorders and related to sleep disorders. Specifically, circulating melatonin was found to be reduced in patients with early PD [225], with blunted cycles in those with excessive daytime sleepiness [226]. Furthermore, PD patients exhibited hypothalamic atrophy, likely involving the suprachiasmatic nuclei, and reduced melatonin output over 24 h was correlated to hypothalamic gray matter volume loss [227].

Importantly, [^18^F]FDOPA PET uptake was reduced in the pineal gland and hypothalamus in patients with advanced PD, possibly related to intrinsic amine synthesis for the pineal gland [228], whereas it was preserved in PD patients with Parkin mutation and in non-symptomatic single parkin mutation carriers [229], who may also have less frequent sleep-related non-motor symptoms [230]. Furthermore, increased [^18^F]FDOPA uptake was observed in patients with early PD [228,231], possibly indicating early compensatory mechanisms, especially in young-onset PD patients.

### 4.8. Ventral Striatum

#### 4.8.1. Anatomy and Function

The ventral striatum is phylogenetically older than the neostriatum comprising putamen and caudate. The ventral striatum consists out of the olfactory tubercle and the accumbens. The latter is located in direct continuity with the caudate and the putamen and can be further subdivided into core and shell. The nucleus accumbens is viewed as a signal integration site, based on its diverse afferents, which stem from the hippocampus (contextual information), the amygdala (emotional information), the prefrontal cortex (glutamatergic, executive and cognitive information) and the midbrain (dopaminergic, motivational significance). In turn, its efferents project to the pallidum, hypothalamus, midbrain and cortical areas; brain regions involved in behavior initiation and complex executive functions [232,233]. Due to its connections, Mogenson et al. define the accumbens as a functional interface between the limbic and the motoric system leading from motivation to action [18]. Taking into account its phylogeny, the nucleus accumbens is considered to be important for the biological drives of survival and reproduction [234]. The reinforcing effect of drugs depends principally on dopaminergic signaling in this brain region [235], hence the accumbens can be seen as the key player of the dopaminergic reward system. Nevertheless, labeling the accumbens just as a reward center is reductionist. Floresco concludes that the accumbens is important for action selection facilitating goal-directed behavior. More precisely, the core mediates approaching to relevant motivational stimuli, whereas the shell suppresses irrelevant actions [232].

#### 4.8.2. Pathology

In Braak staging, the accumbens or the ventral striatum are not directly mentioned, but other nuclei of the basal forebrain are severely affected in Braak stage 4 [28]. However, in this context, it is worth mentioning that Lewy Body pathology is not related to dopaminergic cell loss in the striatum, which indicates that Lewy Body pathology in striatal regions might not be an adequate marker for disease progression and symptom severity [236].

#### 4.8.3. Neuroimaging Evidence in PD

##### Neuropsychiatric Symptoms

Commonly in PD, the accumbens is referred to the pathophysiology of ICD under dopamine replacement therapy. PET imaging of the dopaminergic system revealed that ICDs are associated with a reduction of D2/D3 receptor availability [237,238], dopamine synthesis capacity [146], and DAT density [239] in this region. These “hypodopaminergic” changes of the accumbens are critically involved in ICD and were previously embedded in a vulnerability-stress model for the development of ICD. In this model, a hypodopaminergic state in the accumbens (vulnerability) combined with dopamine replacement therapy (stress) leads to the development of ICD [240]. Task-based fMRI designs shed further light on the involvement of the accumbens in reward learning in ICD: lower BOLD activity was associated with higher subjective value of a delayed reward in hypersexuality [148], and there was a stronger BOLD activity during the initial versus final periods of negative feedback during a gambling task [241]. However, an fMRI paradigm with inhibitory framing and sexual cues could not detect BOLD changes in the accumbens of hypersexual PD patients during a pilot study [242]. Several studies examined connectivity changes of the accumbens in ICD. Whereas larger bet sizes in a virtual casino were associated with a higher structural connectivity to the prefrontal cortex [243,244], ICD severity *per se* was linked to a reduced functional frontostriatal connectivity [146]. Functional connectivity between ventral striatum and subgenual cingulate cortex correlated with reward learning but not with learning from punishment [63]. Therefore, connectivity changes of the accumbens are still ambiguous in ICD.

Concerning reward learning in PD *per se*, abnormal BOLD activity was found in the accumbens during reward anticipation when compared to healthy controls [245] and learning correlated with BOLD activity in this area, which was impaired by dopaminergic treatment [246]. Morphological studies reported on the one hand that implicit risk was associated with higher gray matter volume [247], but on the other hand disinhibition was linked to thinning of the accumbens [248].

An increasing number of studies examined the connection between reward learning and motor activity/skills in order to disentangle the link the between motor symptoms in PD and impaired reward processing. Aerobic motor activity or habitual exercises enhanced reward processing in the accumbens in an fMRI paradigm [249,250]. A raclopride-PET design could show that learning of motor skills in PD leads to a compensatory hyperactivation of the ventral striatum and caudate as compared to healthy controls. However, the controls showed increased dopamine levels in the putamen [251]. Therefore, it seems that motor activity alters reward processing in the accumbens and motor learning itself is processed by the accumbens.

Not only ICDs and learning, but also neuropsychiatric symptoms with reduced impetus such as apathy, depression and anxiety are associated with alterations of the accumbens. There was an inverse correlation between DAT availability and severity of depression [252]. Apathy in PD was associated with reduced amplitude of low-frequency fluctuations as compared to PD controls indicating lower neuronal activity in this brain region [155]. Atrophy of the nucleus accumbens was also reported in patients with apathy [163]. Moreover, apathy was also linked to amyloid deposition in the bilateral accumbens [157].

##### Cognitive and Other Symptoms

Other non-motor symptoms (e.g., pain, sleep, cognition) also go along with a reduced function of the accumbens: the perception of pain under on and off conditions was referred to functional connectivity changes of the accumbens to the motor and sensory cortex [253]. PD patients with sleep disturbance had a lower availability of SERT in the accumbens when compared to PD patients without sleep disturbance [138], and nocturnal hallucinations were related to a reduced volume of this region [90]. Working memory, frontal executive and visuospatial functions were positively correlated with DAT availability in the accumbens [254]. PD patients with MCI and amyloid depositions showed lower DAT density in this area as compared to PD controls [255]. The severity of autonomic dysfunction correlated negatively with DAT density in this region [256].

Other studies examined general neuroimaging changes of the accumbens in PD during the course of disease such as reduction in VMAT2 density [257], lower gray matter volume [57,258] and a lower orientation dispersion of the amygdala accumbens pathway [62]. In an MRI-study, the volume of the ventral striatum was reduced in later-disease stages of PD as compared to earlier disease-stages. Therefore, the authors conclude that volumetric changes of the ventral striatum might serve as a marker of disease progression [259].

##### Conclusions

As a conclusion, imaging changes of the accumbens are associated with the typical neuropsychiatric symptoms in PD such as ICD/impaired reward learning, apathy and depression. Despite of the opposing clinical presentation of these symptoms, in the majority, they seem to go along with a vulnerability state of the accumbens.

## 5. Discussion

The limbic system, a collection of brain structures involved in the processing of emotion and memory, demonstrates marked changes during PD. As outlined above, there is little consensus among researchers on how to precisely define the limbic system, not least on account of its versatile functions. This issue has been a controversial and much disputed subject and has dominated the field for many years, but recent developments offer a new perspective. In this respect, Rolls [11] provides a new framework by postulating not a single but two separate, closely linked limbic systems: the emotion- and memory-centered limbic system.

A useful, operational definition of emotions is that emotions are states associated with stimuli that are either rewarding or punishing and thus emotions are important internal signposts which guide our behavior [260]. The clinical picture of PD is dominated by emotion-related non-motor symptoms such as depression, anxiety, apathy, and ICD. These symptoms place a severe burden on the patient and their caregivers. Therefore, recent neuroimaging evidence has been of critical importance to highlight major contributions of disease-related damage to the amygdala, hippocampus, ventral striatum, and cingulate gyrus to these affective symptoms. According to Rolls, the neural basis of emotions can be divided into three tiers. First, information processing starts at a level at which neurons encode ‘what’ the input or stimulus represents, independent of its value. This step mainly involves the primary sensory cortices. Second, the value of the stimulus is computed. In this tier, the limbic system comes into play, mainly involving the amygdala. The amygdala as well the OFC (not traditionally seen as part of the limbic system) are implicated in holding representations of stimulus value, learning new reinforcement associations and updating such associations when contingencies change. After the stimulus value has been computed in tier two, brain structures of the final third tier (ACC, medial prefrontal cortex, hypothalamus, basal ganglia) are involved in decision making between stimuli of different value, selecting an appropriate behavior as well as action-outcome learning.

Regarding PD symptomatology, it is important to consider that the connectivity of the emotion system is primarily feedforward—from tier one to three. In this sense, the OFC has projections to the ventral striatum, caudate nucleus, ACC, medial prefrontal cortex, and hypothalamus. Some of these connections represent pathways particularly important for the production of behavior. A great body of evidence (which has been reviewed in detail above) suggests alterations of these routes in PD. For example, it has been reported that damage in the OFC can cause failure to compute and update the reward value of stimuli and thus might underly emotion impairments, such as lack of affect, irresponsibility, and impulsivity. In PD, ICD (excessive urges and behaviors including pathological gambling, binge eating, hypersexuality, and compulsive buying [261]) is a commonly reported symptom and has been strongly linked to the alterations of processing in hubs as the ventral striatum and cingulate gyrus. In this regard, it has been shown that the connectivity strength of these two regions seems to be negatively correlated with the severity of impulsivity and, furthermore, the severity of impulsivity is also associated with a thicker cingulate cortex [146]. Moreover, ICD is inter alia associated with an altered connectivity between the ventral striatum and prefrontal cortex [243]. Besides the direct disease related alteration of limbic regions, deficient processing on the level of tier two being transmitted to tier three brain regions may also contribute to malfunction and manifestation of PD symptoms such as apathy, depression and anxiety.

In addition, the computation of reward prediction errors (difference between expected and actual reward), which is impaired in PD, is associated with dopaminergic neurons of the midbrain, and computation in the tier two brain regions, whereby tier two output regions represent brain systems concerned with action performance. Now, interestingly, it is hypothesized that PD-motor symptoms may be related to a shift in the cost–benefit computation, downweighting the expected reward [262,263]. In this regard, a study which employed a physical force task found that PD induced dopamine depletion reduced the amount of effort PD patients were willing to produce for a given reward [264]. This further supports the hypothesis of limbic pathology contributing to the cardinal PD motor symptoms, involving the emotion-centered limbic system.

In line with Rolls, it is notable that damage to the emotion-centered limbic system does not severely affect episodic memory or the processing of spatial information, the main functions of the memory-centered limbic system. The amygdala, as part of the emotional limbic system, demonstrates strong connections with the ACC, whereas the hippocampus, the main structure of the memory limbic system, exhibits mayor connections with the PCC, which in turn is connected to areas involved in spatial functioning, including the visual parietal cortex, supporting the existence of two separate limbic systems. However, the systems are not independent of each other, as the hippocampus does receive a signal from reward processing areas such as the OFC and amygdala via the entorhinal and perirhinal cortex.

Dysfunction of the memory-centered limbic system in the form of hippocampal damage, characterized by impaired episodic memory, is observed in many PD patients. In this sense, increased disease duration is strongly linked to cognitive decline, manifesting as dementia in late-stage PD [79]. Neuroimaging evidence suggests that hippocampal volume decreases as the disease advances, whereby the severity of volume loss is a predictor for conversion to dementia [53,98,99]. Additionally, hippocampal cholinergic innervation is also strongly depleted in PD dementia [76]. Now, considering the second major function of the memory-centered limbic system, the processing of spatial information, it has been found that PD related hippocampal alterations are associated with impaired spatial working memory, which can be explained by the essential role of the hippocampus and its connections in object-place memory [95].

Overall, PD pathology is prominent in the limbic system throughout the disease course, responsible for disabling nonmotor, neuropsychiatric, behavioral, and cognitive symptoms. Although the pathophysiology remains complex, neuroimaging helps disentangling specific network injury, supporting the operational dichotomy between memory-centered and emotion-centered limbic systems and related symptoms [11]. Additionally, due to the increasing use of imaging techniques with high spatial resolution such as 7T MRI, more information will also be gained concerning the small limbic structures. Indeed, the two limbic systems seem to operate in an independent fashion, although neuroimaging in PD patients highlights the network organization and critical role of the ventral striatum and ACC and their modulation by brainstem and forebrain small nuclei.

To conclude, alterations of the limbic system play an important role in PD symptomatology, including affective, but also cognitive and motor symptoms. Particularly, the latter aspect has not yet received adequate attention and highlights how motor and nonmotor symptoms are deeply intertwined in PD.

## Data Availability

Not applicable.

## Supplementary Materials

The following supporting information can be downloaded at: https://www.mdpi.com/article/10.3390/brainsci12091248/s1, Supplementary Table S1.
